# A Systematic Review and Meta-analysis of Ventilator-associated Pneumonia in Adults in Asia: An Analysis of National Income Level on Incidence and Etiology

**DOI:** 10.1093/cid/ciy543

**Published:** 2018-07-05

**Authors:** Ana Bonell, Ryan Azarrafiy, Vu Thi Lan Huong, Thanh Le Viet, Vu Dinh Phu, Vu Quoc Dat, Heiman Wertheim, H Rogier van Doorn, Sonia Lewycka, Behzad Nadjm

**Affiliations:** 1 Oxford Clinical Research Unit Hanoi, National Hospital for Tropical Diseases, Vietnam; 2 University of Miami Miller School of Medicine, Florida; 3 National Hospital for Tropical Diseases; 4 Hanoi Medical University, Vietnam; 5 Department of Medical Microbiology, Radboud Center of Infectious Diseases, Radboud University Medical Center, Nijmegen, The Netherlands; 6 Nuffield Department of Medicine, University of Oxford, United Kingdom

**Keywords:** ventilator-associated pneumonia, review, Asia, antibiotic resistance

## Abstract

**Background:**

Ventilator-associated pneumonia (VAP) is the commonest hospital-acquired infection (HAI) in intensive care. In Asia, VAP is increasingly caused by resistant gram-negative organisms. Despite the global antimicrobial resistance crisis, the epidemiology of VAP is poorly documented in Asia.

**Methods:**

We systematically reviewed literature published on Ovid Medline, Embase Classic, and Embase from 1 January 1990 to 17 August 2017 to estimate incidence, prevalence, and etiology of VAP. We performed a meta-analysis to give pooled rates and rates by country income level.

**Results:**

Pooled incidence density of VAP was high in lower- and upper-middle-income countries and lower in high-income countries (18.5, 15.2, and 9.0 per 1000 ventilator-days, respectively). *Acinetobacter baumannii* (n = 3687 [26%]) and *Pseudomonas aeruginosa* (n = 3176 [22%]) were leading causes of VAP; *Staphylococcus aureus* caused 14% (n = 1999). Carbapenem resistance was common (57.1%).

**Conclusions:**

VAP remains a common cause of HAI, especially in low- and middle-income countries, and antibiotic resistance is high.

## Teaser

Ventilator-associated pneumonia (VAP) is the commonest infection in intensive care. Our systematic review and meta-analysis found VAP incidence rates to be highest in low-income compared with high-income countries in Asia. Antimicrobial resistance rates are also alarmingly high.

Ventilator-associated pneumonia (VAP) is the single most common hospital-acquired infection (HAI) in intensive care units (ICUs) around the world [1, 2]. Patients who develop VAP have higher mortality, longer hospital stays, higher antibiotic usage, and more costly treatment than those without VAP [3–6].

Many low- and middle-income countries in Asia have rapidly developing economies with increasingly sophisticated provision of healthcare, resulting in more people accessing healthcare [7]. This, combined with a lack of regulation or enforcement on antibiotic dispensing, underdeveloped antibiotic stewardship, and infection prevention and control programs, has led to a crisis in antibiotic resistance in hospital and community settings [8, 9]. In the hospital setting, ICUs often have the highest levels of antibiotic resistance as a result of dealing with severely ill patients with high levels of HAI and antibiotic usage. VAP itself is associated with pathogens with high levels of antibiotic resistance, resulting in the need to treat with broad-spectrum antibiotics, which further drives antibiotic resistance [10–12].

In high-income countries, a combination of surveillance, education, and tailored intervention and prevention bundles have led to a reduction in the incidence of VAP [13]. In Asia there are limited data on rates of VAP, the common associated pathogens, and their antibiotic susceptibility profiles. This systematic review and meta-analysis of VAP incidence, prevalence, microbiological etiology, and cost summarizes these data by country income level and informs prevention and intervention guidelines and further research in the region.

## METHODS

**Figure 1.**
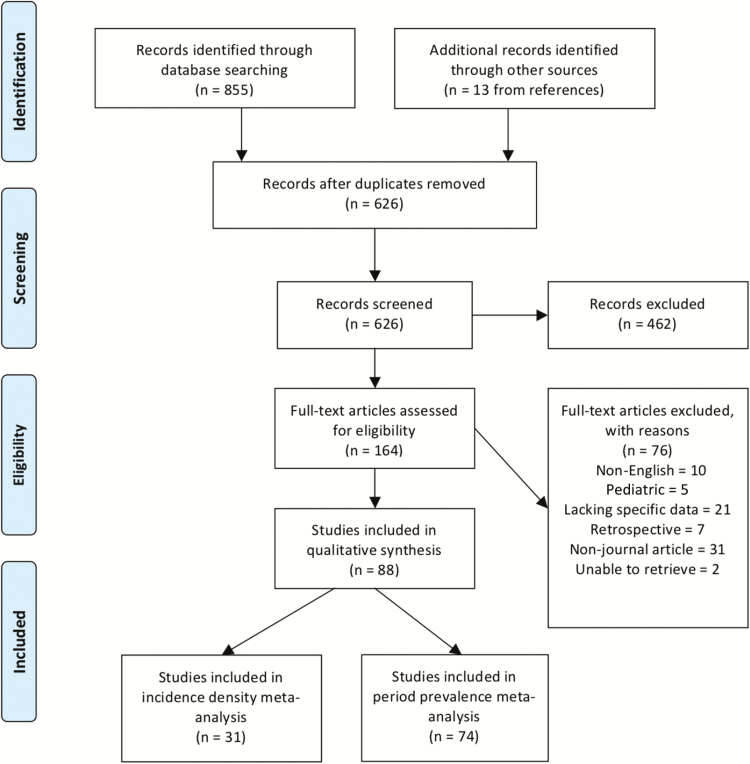
Preferred Reporting Items for Systematic Reviews and Meta-Analyses (PRISMA) 2009 flow diagram [14].

The systematic collection of data followed the Preferred Reporting Items for Systematic Reviews and Meta-Analyses (PRISMA) guidelines. A literature search of 2 databases, Embase and Ovid, was performed from 1 January 1990 to 17 August 2017. The search strategy was ventilator-associated pneumonia (meshed and unmeshed terms) AND Asia OR Afghanistan, Bahrain, Bangladesh, Bhutan, Brunei, Cambodia, China, Hong Kong SAR China, India, Indonesia, Iran, Iraq, Japan, Jordan, Kazakhstan, Kyrgyzstan, Kuwait, Laos, Lebanon, Macau SAR, Malaysia, Mongolia, Myanmar, Nepal, North Korea, Oman, Pakistan, Qatar, Saudi Arabia, Singapore, South Korea, Sri Lanka, Syria, Taiwan, Tajikistan, Thailand, The Philippines, Turkmenistan, Uzbekistan, United Arab Emirates, Vietnam, Yemen. Articles were included for full screening if the abstract indicated there was prospective data on etiology, incidence density, period prevalence, or any details on the economic cost of VAP (see Figure 1 for details regarding inclusion of studies). Two independent reviewers assessed the titles and abstracts of papers for inclusion and any discrepancies were referred to a third independent reviewer. References of papers included were also assessed and included if they fit the inclusion criteria. Data were extracted onto a standardized form that was trialed on the first 3 papers and implemented after minor adjustments. Studies were categorized as those that gave period prevalence data (also referred to as cumulative incidence, defined as the number of episodes of VAP per 100 patients over a period of time, usually their ICU admission), incidence density data (number of episodes of VAP per 1000 ventilator-days, included if the total number of ventilator-days was also given), microbiology data with or without susceptibility, and any data on costing of VAP. Microbiology data were only included when the total number of organisms was given. Antibiotic resistance was classified using the European Centre for Disease Prevention and Control guidelines on surveillance of HAIs in the ICU [15]. Cost of VAP was collected in US dollars where possible and converted from local currency at the average rate for the year data was collected in the study. Study quality was assessed using the Strengthening the Reporting of Observational Studies in Epidemiology (STROBE) checklist for observational cohort and case-control studies, and the Consolidated Standards of Reporting Trials (CONSORT) checklist was used for randomized trials. Grading was determined by percentage completion of the checklist; high, ≥80% of items; moderate, 50%–80%; poor, <50% [16].

Countries were defined according to World Bank classification of income level as low income (LIC), lower-middle income (LMIC), upper-middle income (UMIC), and high income (HIC) at the time the study was published [17]. There were only 2 studies from LICs (both from Nepal) and therefore these were included with LMICs for all group analysis. Countries were defined as tropical or nontropical based on their geographical latitude.

Data were analyzed using Stata software version 14 and Comprehensive Meta-analysis Software (www.meta-analysis.com). Data were pooled in all studies that reported the same outcome measures. The outcome measures of interest were incidence density as rate of VAP per 1000 ventilator-days with total number of ventilator-days as a denominator; period prevalence as rate of VAP per 100 patients over a given period of time; total number of microorganisms identified per country and proportion of individual species; and antibiotic resistance rates for a species as percentage of resistant organisms of that species by total number of organisms of that species identified per country. Meta-analysis was conducted on incidence density and period prevalence by weighted average estimates from individual studies. Forest plots were generated by Stata and meta-regression was used to analyze comparable groupings—country, income level, year published, study quality, study method (randomized controlled trials, observational cohort, pre-/postintervention, quasi-experimental), hospital type (specialist, tertiary/university, district, multiple), and ICU type (general, medical, surgical, neurological, trauma, coronary, multiple). Studies were assessed for heterogeneity using *I*^2^ statistic and χ^2^ test (taking *P* < .05 to indicate statistically significant heterogeneity). When significant heterogeneity was found, then random-effects meta-analysis was used [18]. A funnel plot was used to determine the probability of publication bias. Comparisons of rates were analyzed using mixed effect meta-regression, an unrestricted maximum likelihood model.

Microbiological identification and susceptibility data were individually pooled by country and summary measures presented without use of meta-analysis.

## RESULTS

A total of 88 studies covering 22 countries were included in the final data extraction. One hundred sixty-four were identified for full article review, and 76 were excluded for the following reasons: non-English language (n = 10), pediatric (n = 5), lacking specific data (n = 21), retrospective (n = 7), non–journal article (n = 31), and unable to retrieve (n = 2) (Figure 1). The majority of studies were published in the last 10 years (2008 onward, 67/88 [76%]). There were 69 (78%) studies of high quality, 19 (22%) of moderate quality, and none of poor quality. Data on VAP incidence density were available from 31 articles and period prevalence from 74 articles. Microbiology data were available from 70 studies, of which 49 also included data on resistance rates (see Supplementary Materials for full list of studies). The total number of patients in all studies was 564355, and the total number of ventilator-days was 1079309 with 21176 episodes of VAP. The median age of patients was 52.0 years (interquartile range, 40.2–57.6 years) and sex was available for 141354 patients, of whom 93108 were male (65.9%).

### Incidence Density and Prevalence Data

Data on incidence density were available from 14 countries, which included 538600 patients, 1076140 days of mechanical ventilation, and 16082 episodes of VAP. The majority of these studies were published in the last 10 years (26/31 [84%]). Significant heterogeneity was observed in the studies as a whole (*I*^2^ = 100%) and when the studies were divided both by country and by income group (*I*^2^ approximately 99% in all categories). Therefore, all analysis was done using the random-effects model. The point estimate incidence density of VAP/1000 ventilator-days in all studies was 15.1 (95% confidence interval [CI], 12.1–18.0). Meta-analysis of incidence density per income level is presented in Table 1. There was a higher incidence density rate in LMICs compared with HICs when analyzed by meta-regression (18.5 vs 9.0 per 1000 ventilator-days; *P* = .035). Meta-analysis of studies by study quality, study type, year of publication, hospital type, and ICU type did not show any significant difference in incidence density or prevalence (see Supplementary Materials for full analysis).

**Table 1.** Incidence Density and Prevalence of Ventilator-associated Pneumonia From Meta-analysis Analyzed by Country Income Level

Pooled Incidence Density Rate (per 1000 VD)		No. of Studies	(95% CI)	*I* ^2^	*P* Value for Heterogeneity
LIC/LMIC	18.5	14	(15.6–21.4)	99.99	<.001
UMIC	15.2	9	(9.3–21.0)	99.99	<.001
HIC	9.0	8	(6.8–11.2)	99.99	<.001
Total	15.1	31	(12.1–18.0)	100.00	<.001
Pooled Period Prevalence Rate, %					
LIC/LMIC	13.3	35	(8.6–20.2)	99.28	<.001
UMIC	15.4	24	(9.5–24.1)	99.67	<.001
HIC	8.0	15	(4.6–13.6)	99.10	<.001
Total	12.7	74	(10.0–16.1)	99.48	<.001

Abbreviations: CI, confidence interval; HIC, high-income country; LIC, low-income country; LMIC, lower-middle-income country; UMIC, upper-middle-income country; VD, ventilator-days.

**Figure 2.**
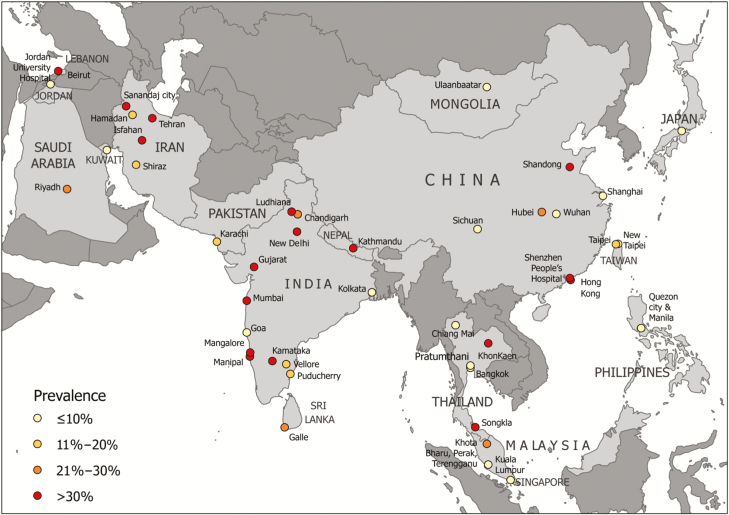
Mapped period prevalence rates of ventilator-associated pneumonia for 74 studies in Asia. The prevalence rates reported are color coded. Source: http://naturalearthdata.com.

The pooled period prevalence of VAP for all studies was 12.7% (95% CI, 10.0–16.1). This includes 18400 episodes of VAP and 549478 patients. Meta-analysis of period prevalence per income level is presented in Table 1 (see Supplementary Materials for full analysis). Again, analysis by study quality, study type, year of publication, hospital type, and ICU type did not show any significant difference. Period prevalence was highest in UMICs and lowest in HICs. Pooled incidence density and pooled prevalence data per country are presented in Table 2, and prevalence by country and region in Figure 2. The highest pooled incidence density was found in Mongolia (43.7/1000 ventilator-days) and the highest pooled prevalence in Hong Kong (48.1%), each based on 1 study [19, 20].

**Table 2.** Pooled Results From Meta-analysis by Country, Incidence Density, and Period Prevalence

Country, by Income Level	Total No. of Studies	Total No. of VD	Incidence Density per (1000 VD)	(95% CI)	Total No. of Studies	Period Prevalence, %	(95% CI)
LIC/LMIC							
Nepal	1	3364	21.4	(21.2–21.6)	2	21.7	(9.4–42.4)
India	7	106668	16.6	(12.5–20.7)	23	14.3	(7.5–25.8)
Mongolia	1	618	43.7	(43.2–44.2)	1	5.8	(4.0–8.3)
Pakistan	…	…	…	…	1	15.5	(12.7–18.6)
Philippines	1	10041	16.7	(16.6–16.8)	1	4.3	(3.6–5.1)
Sri Lanka	…	…	…	…	1	26.5	(17.4–38.2)
Jordan	…	…	…	…	1	8.1	(7.0–9.2)
UMIC							
China	6	628193	19.0	(12.1–25.9)	9	11.4	(5.1–23.5)
Iran	1	6976	7.9	(7.8–7.9)	10	18.2	(9.3–32.5)
Lebanon	1	3561	8.1	(8.0–8.2)	2	16.7	(1.1–78.8)
Malaysia	…	…	…	…	2	12.6	(2.3–47.6)
Thailand	5	43692	8.8	(6.6–11.0)	6	13.2	(5.6–28.2)
HIC							
Hong Kong	…	…	…	…	1	48.1	(30.4–66.4)
Japan	1	51361	12.6	(12.6–12.6)	1	2.9	(2.7–3.2)
Kuwait	2	17465	8.8	(8.1–9.4)	2	2.5	(1.0–6.0)
Saudi Arabia	2	32988	3.6	(1.7–5.4)	4	11.1	(5.2–22.2)
Singapore	…	…	…	…	1	8.1	(4.5–14.0)
Taiwan	2	94464	10.1	(5.0–15.2)	6	7.9	(2.3–23.9)
3 Gulf countries^a^	1	76749	4.8	(4.8–4.8)	…	…	…

Abbreviations: CI, confidence interval; HIC, high-income country; LIC, low-income country; LMIC, lower-middle-income country; UMIC, upper-middle-income country; VD, ventilator-days.

aBahrain, Oman, Saudi Arabia.

### Microbiology

A total of 14295 organisms were identified. The most common organism was *Acinetobacter baumannii* (n = 3687 [26%]), followed by *Pseudomonas aeruginosa* (n = 3176 [22%]), *Klebsiella pneumoniae* (n = 2008 [14%]), and *Staphylococcus aureus* (n = 1999 [14%]).

**Figure 3.**
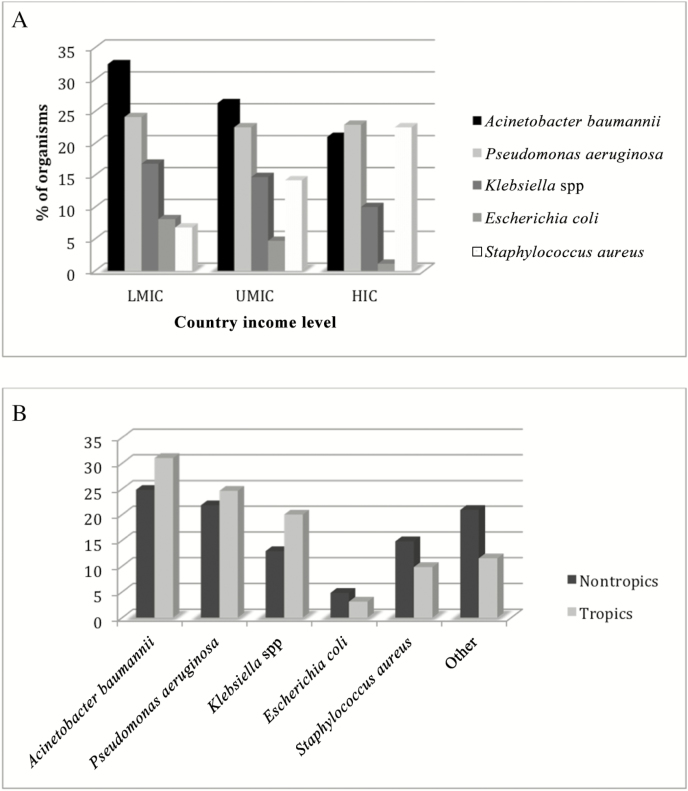
*A*, Pooled microbiology results of ventilator-associated pneumonia (VAP) etiology by country income level. *B*, Pooled microbiology results of VAP etiology by geographic area (tropical vs nontropical). Abbreviations: HIC, high-income country; LMIC, low-/lower-middle-income country; UMIC, upper-middle-income country.

**Figure 4.**
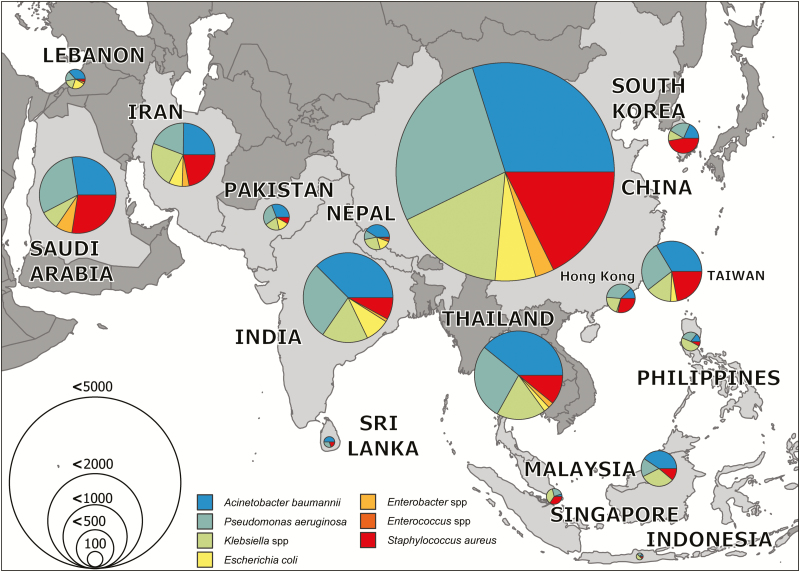
Mapped pooled microbiology results by country for etiology of ventilator-associated pneumonia. Organisms are color coded, and the size of the pie chart reflects the total number of isolates identified per country. Source: http://naturalearthdata.com.

When the data were split by income level, *A. baumannii* was the most common organism in the LMIC group. However, the proportion due to this organism gradually reduced as income levels increased, and *S. aureus* and *P. aeruginosa* were the most common in the HIC group (Figure 3A). When the studies were divided by geographical latitude into tropical and nontropical regions, *A. baumannii* was more common in the tropical regions whereas *S. aureus* was a more common cause of infection in nontropical regions (Figure 3B). Aggregated microbiology data are presented by country for the top 7 organisms in Figure 4.

### Antimicrobial Resistance

Antimicrobial susceptibility of *A. baumannii* was available from 34 studies, including a total of 1230 organisms tested. Carbapenem resistance was found in 702 isolates (57.1%). When grouped by income level, the resistance rate was 58% (199/342), 65% (457/706), and 26% (47/182) for LMICs, UMICs, and HICs, respectively. When divided into tropical and nontropical regions, the carbapenem resistance rate was 77.9% (307/394) and 47.3% (395/836), respectively.

Antimicrobial susceptibility of *P. aeruginosa* was available from 35 studies, which included 1124 organisms. Carbapenem resistance was found in 414 isolates (36.9%). When grouped by income level, resistance was 41% (88/213), 42% (298/705), and 14% (28/206) in LMICs, UMICs, and HICs, respectively. Geographically, the carbapenem resistance rate among *P. aeruginosa* in the tropics was 43% (167/388) compared with 34% (247/736) in the nontropical regions.

Resistance data on *S. aureus* were available from 38 studies, including 864 organisms. Overall, the proportion of *S. aureus* that was methicillin resistant (MRSA) was 67% (577/864), with 50% (26/50) in LMICs, 67% (331/493) in UMICs, and 69% (220/319) in HICs. MRSA was equally common among *S. aureus* in the tropical and nontropical regions (64% [112/173] and 67% [466/691], respectively).

Antimicrobial susceptibility of *K. pneumoniae* to third-generation cephalosporins (625 organisms) and carbapenems (638 organisms) were available from 30 studies. Cephalosporin resistance was found in 370/625 (59%) isolates and carbapenem resistance in 101/638 (15.8%) isolates.

Antimicrobial susceptibility of *Escherichia coli* to third-generation cephalosporins (165 organisms) and carbapenem (152 organisms) were available from 22 and 19 studies, respectively. Cephalosporin resistance was found in 127/165 (77%) isolates and carbapenem resistance in 41/152 (27%) isolates.

### Cost Burden of VAP

There were very few data available on the costs of VAP in Asia. Only 5 studies in our review included any information on the economic burden of VAP and these used different methodologies and therefore could not be compared. Three studies gave ICU costs of patients with and without VAP. In 2011–2012, in Ludhiana, India, it was estimated that the average cost of an ICU admission for a patient with VAP was $6250.92 compared with $2598.84 for a patient without VAP [21]. In Tehran, Iran, in 2012–2013, VAP cost 3.04 purchasing power parity (PPP) per day, compared with 1.23 PPP per day for those without VAP [22]. In Taipei, Taiwan in 2011–2014, an average ICU admission with VAP cost $8924.50 compared with $6625.50 without [23]. One further study in 2011–2012 in Goa, India, calculated the average additional cost of antibiotics used to treat VAP at $432.7 [24]. An additional study estimated the cost benefit of introducing a VAP educational bundle in Thailand and found the average hospital cost per patient admitted to ICU was $4769 prior to the bundle and $2378 after the bundle [25].

## DISCUSSION

VAP remains a major contributor to HAIs in Asia. This systematic review highlights very worrying aspects of the ongoing crisis in HAIs and antibiotic resistance affecting this region. The incidence density of VAP remains high in both LMICs and UMICs, demonstrated by no indication of a reduction over time on meta-analysis. Given the large expansion of ICUs in these settings, the at-risk population can be expected to rise substantially in the immediate future, compounding the problem. Additionally, for LMICs anticipating a resolution of the problem as their economies grow, the data from the UMICs in the region are worrying. There are major omissions in the published data from many of the countries included in this review with only 22 of 40 countries having any published data at all. The changing trend in microbiological etiology from previous published reviews (where *S. aureus* was the predominant microorganism) and the rate of resistance in these organisms reinforces the picture of a global antibiotic crisis [26, 27].

### Incidence Density and Prevalence Data

To our knowledge, this is the first review to analyze incidence data by national income level. We have demonstrated a trend in the incidence density of VAP by income level but no trend in the period prevalence. Our pooled period prevalence has demonstrated that UMICs also have a significant burden of VAP and that this may be higher than LMICs. When comparing with other international VAP studies, the International Nosocomial Infection Control Consortium (INICC) report from 2009 gives a pooled density of 13.6 per 1000 ventilator-days, which includes data from Latin America, Asia, Africa, and Europe [28]. Our overall rate is similar to this; however, when taking the LMIC alone, we have shown much higher rates, similar to those documented in a systematic review of developing countries globally (22.9/1000 ventilator-days) [29].

It is currently complicated to compare national rates of VAP with the rate in the United States due to changing definitions and means of classifying VAP [30]. However, the published rates of 0.0–5.8 per 1000 ventilator-days in the United States are comparable to some but not all of the studies from HICs in this review [31]. Previous reviews have quoted incidence density rates of 2–16 per 1000 ventilator-days (from the INICC and United States), whereas we have found much higher rates in some of our studies (0.50–76.83) [32]. A systematic review and meta-analysis of VAP in China determined the incidence density at 24.14 episodes per 1000 ventilator-days [33]. This is higher than the rate we have found in China (19.0/1000 ventilator-days). Whereas we included 13 articles from China, the aforementioned review included 195 additional studies published in non–English-language journals, suggesting higher rates in these publications.

### Microbiology and Resistance Rates

This review adds to the evidence of *A. baumannii* as the primary organism associated with VAP; it is the commonest organism in LMICs and in countries in the tropics and has high rates of antimicrobial resistance in these regions. This is the first systematic review to collate studies from Asia that demonstrates *A. baumannii* as the main cause of VAP [11, 34]. Other studies have demonstrated other gram-negative bacteria, such as *P. aeruginosa*, as a major cause of VAP [10]. However definitive conclusions are hard to draw since the diagnosis of VAP is complicated and isolated pathogens may be colonizers and not the causative agent.

The resistance rates we have found are similar to those documented by the INICC and others globally and demonstrate a worrying rise in resistance in all pathogens to even the broadest antibiotics (eg, rates of carbapenem resistance in *A. baumannii* at approximately 75%) [35–38]. Resistance rates to colistin were not available in any of the studies. Our review confirms what multiple single-site studies and expert reviews have stated, that resistant gram-negative bacteria are the major cause of VAP in Asia [37, 39].

### Cost Burden of VAP

There is very sparse information on the health economic impact of VAP in Asia. There are several studies describing the disease burden of HAI in Asia or LMIC settings, all of which have found high rates of VAP and a significant increase in mortality and length of stay [16, 29, 40]. Our study aimed to summarize this sparse literature on direct economic burden of VAP, but due to the lack of published data on this we are unable to draw any definitive conclusions. It would appear that VAP is a significant cause of patient morbidity and mortality, that the rates remain high, and that cost of prevention bundles in this setting are both clinically and financially worthwhile.

Our study is limited by the inclusion of only English-language journals. We excluded several studies from our search due to use of a non-English language and we did not search non-English-language databases. In addition, there was a large degree of heterogeneity in our studies, which we have attempted to compensate for by using the random-effects model for meta-analysis, but the underlying differences in the studies remain. These differences result from the wide geographical area covered as well as different practices within the ICU, different patient groups within the ICUs, and different definitions used to diagnose VAP. Due to the publication bias and heterogeneity, any conclusions and extrapolations to other populations should be undertaken with caution.

In conclusion, VAP is a significant burden in Asia, with LMICs and UMICs both demonstrating higher rates than HICs. There is a worrying amount of antibiotic resistance in the pathogens causing VAP and unless concerted international effort is made, this is likely to worsen.

## Supplementary Data

Supplementary materials are available at *Clinical Infectious Diseases* online. Consisting of data provided by the authors to benefit the reader, the posted materials are not copyedited and are the sole responsibility of the authors, so questions or comments should be addressed to the corresponding author.

Prevalence Random Forest Plot Income Level

Incidence Density Random Forest Plot Studytype

Incidence Density Random Forest Plot Hospitaltype

Incidence Density Random Forest Plot Income Level

Metaregression Year on Incidence Density

Additional Forest Plots of Incidence Density

All studies included in SR review

Incidence Density Random Forest Plot Country

Metaregression of Year Published on Prevalence

Incidence Density Random Forest Plot Qualitygrade

Incidence Density Random Forest Plot Icutype

## Notes

***Financial support.*** This work was supported by the Wellcome Trust through the Asia Program Core Grant.

***Potential conflicts of interest.*** All authors: No reported conflicts of interest. All authors have submitted the ICMJE Form for Disclosure of Potential Conflicts of Interest. Conflicts that the editors consider relevant to the content of the manuscript have been disclosed.
