# Supplementary figures and images for: A Systematic Review and Meta-analysis of Ventilator-associated Pneumonia in Adults in Asia: An Analysis of National Income Level on Incidence and Etiology
Source: Clin Infect Dis. 2018 Jul 5;68(3):511–8. doi: 10.1093/cid/ciy543 (PMC6336913; doi:10.1093/cid/ciy543)

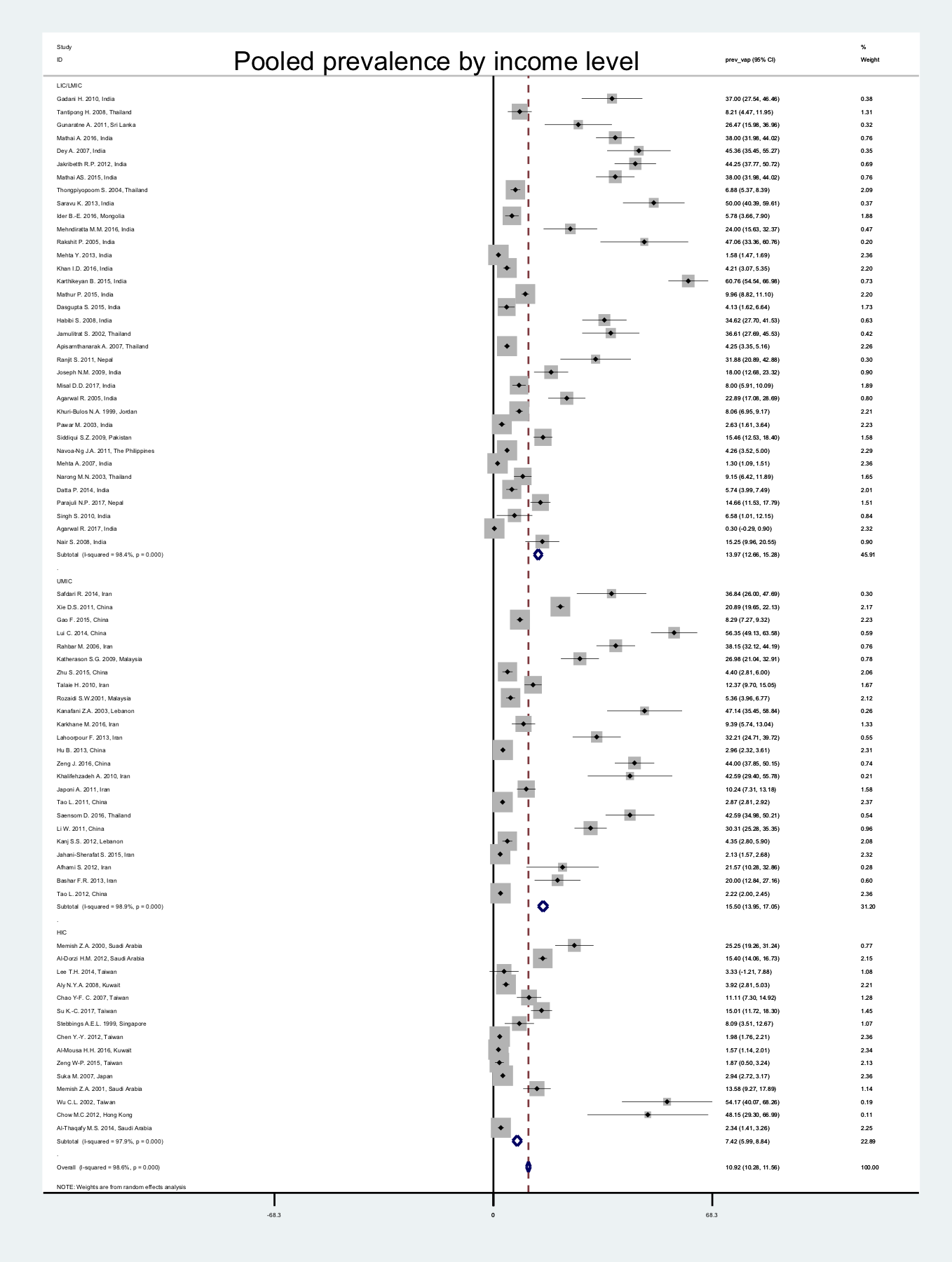

Supplement: Prevalence Random Forest Plot Income Level [file ciy543_suppl_prevalence_random_forest_plot_income_level.png]

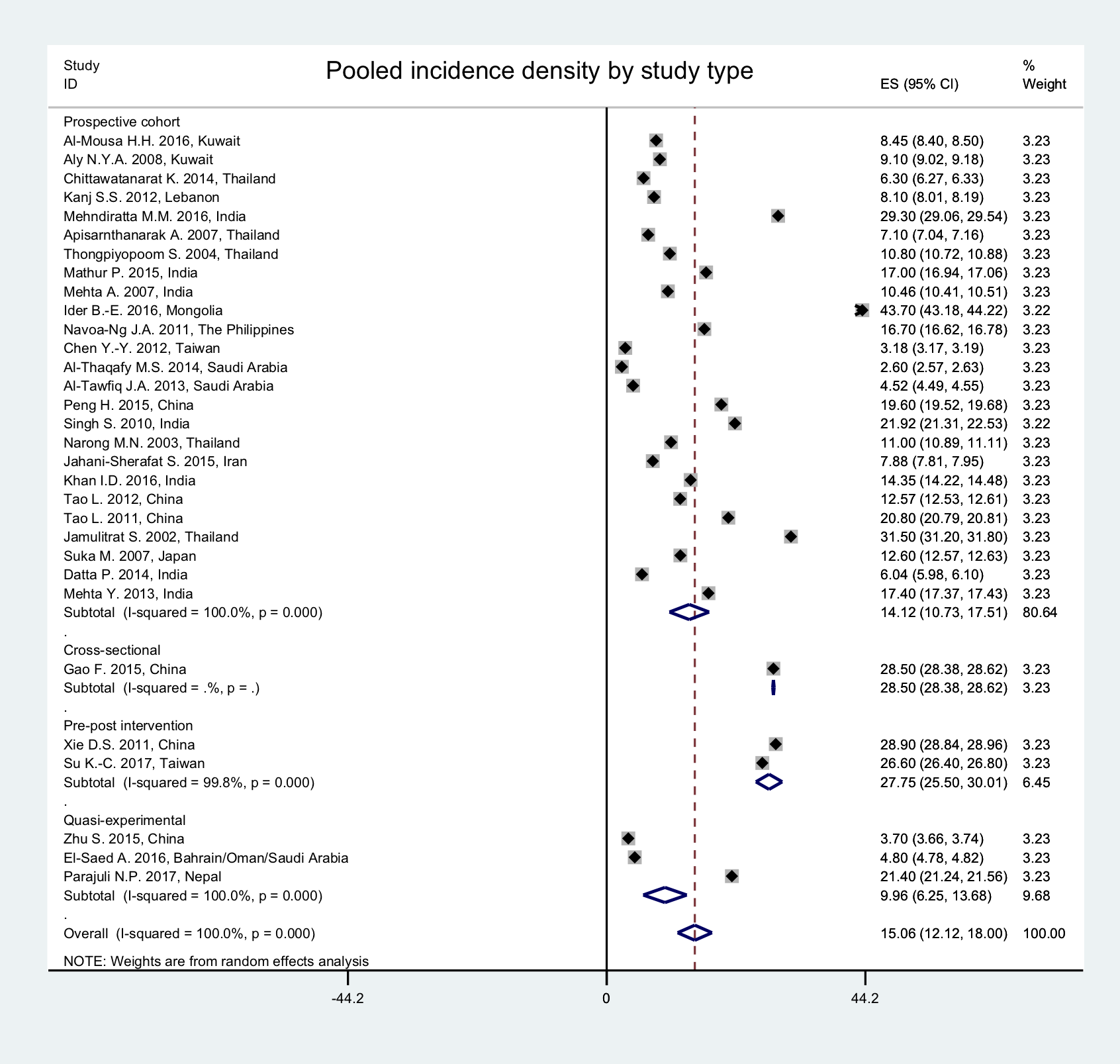

Supplement: Incidence Density Random Forest Plot Studytype [file ciy543_suppl_incidence_density_random_forest_plot_studytype.png]

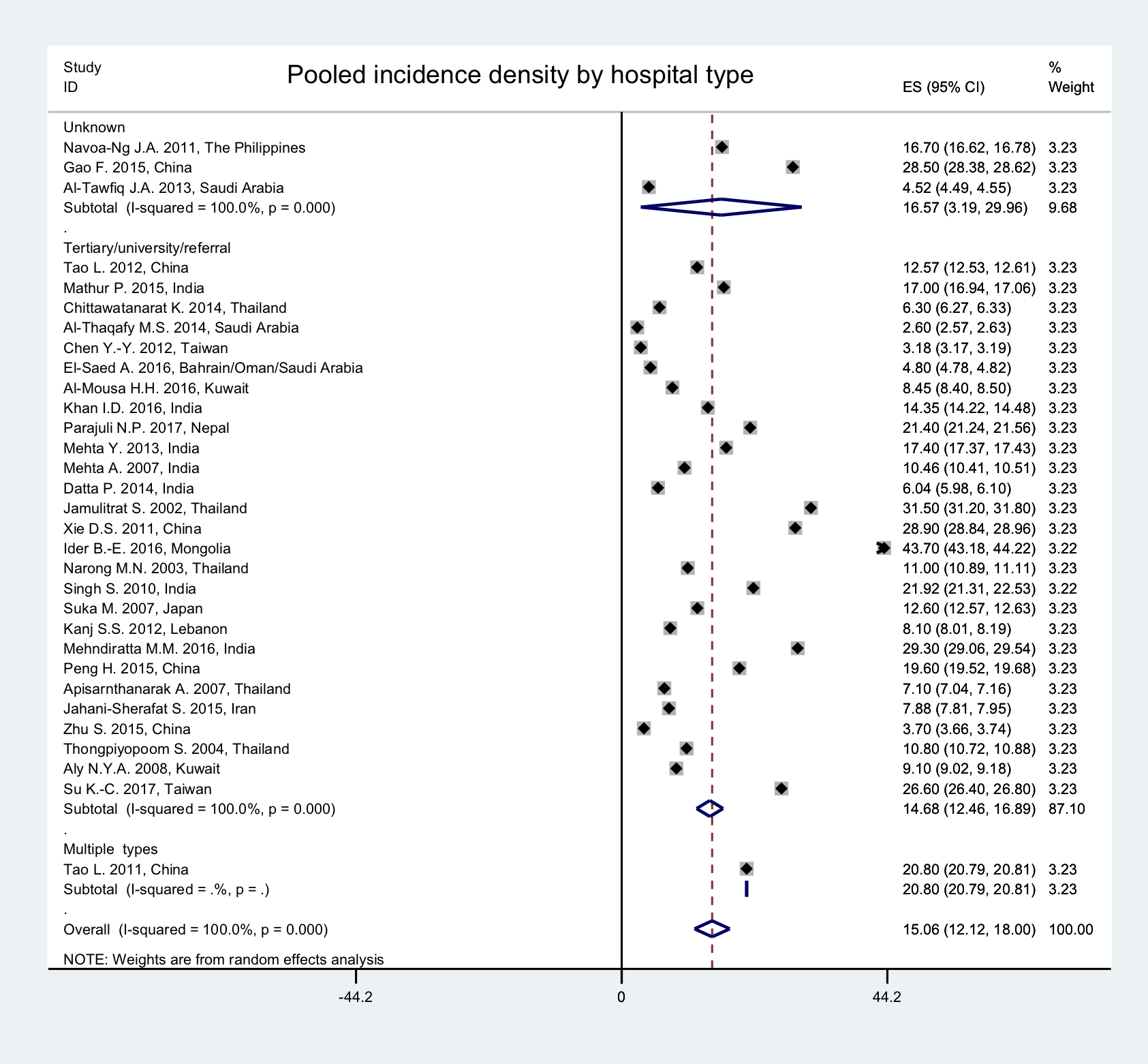

Supplement: Incidence Density Random Forest Plot Hospitaltype [file ciy543_suppl_incidence_density_random_forest_plot_hospitaltype.png]

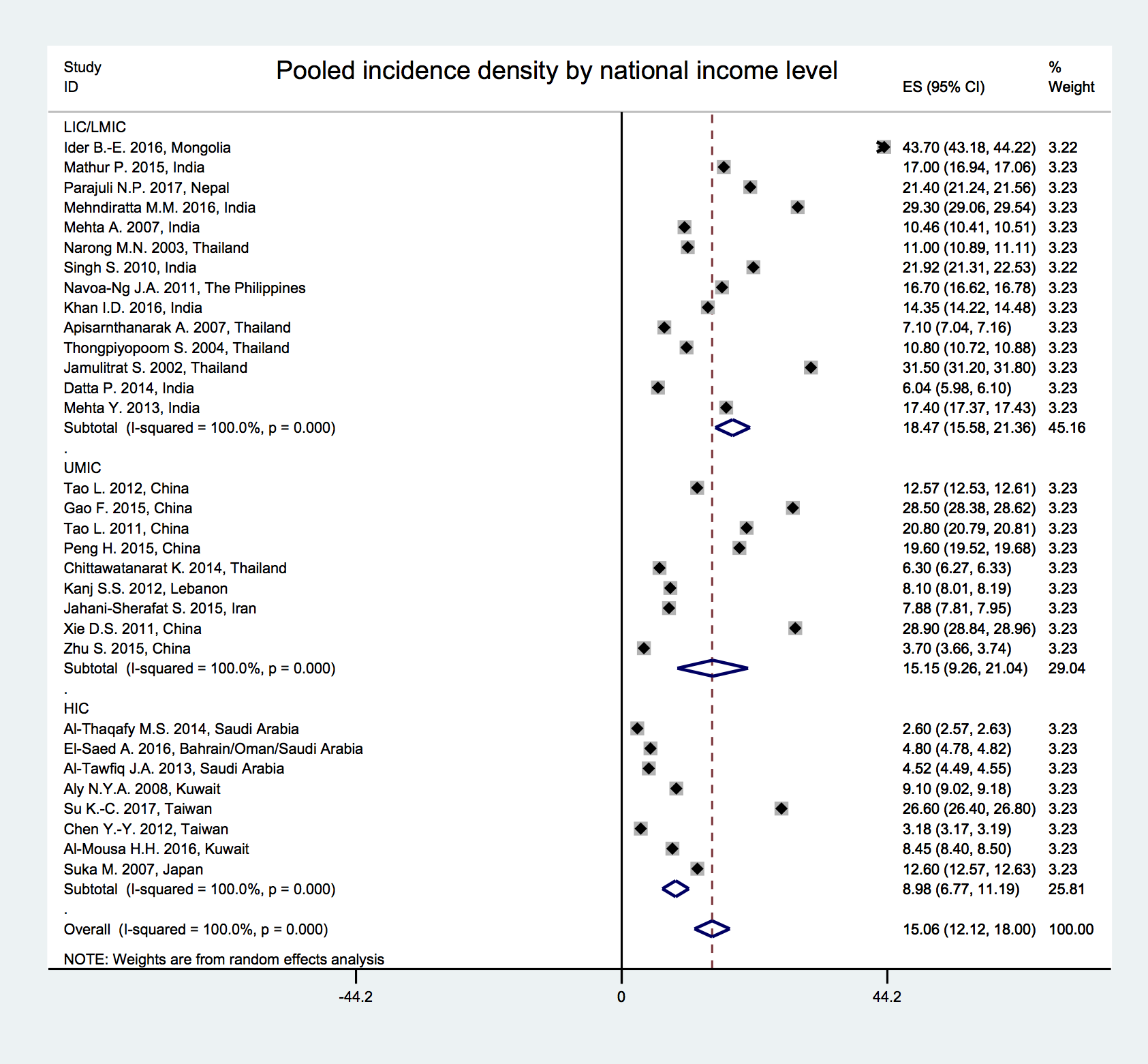

Supplement: Incidence Density Random Forest Plot Income Level [file ciy543_suppl_incidence_density_random_forest_plot_income_level.png]

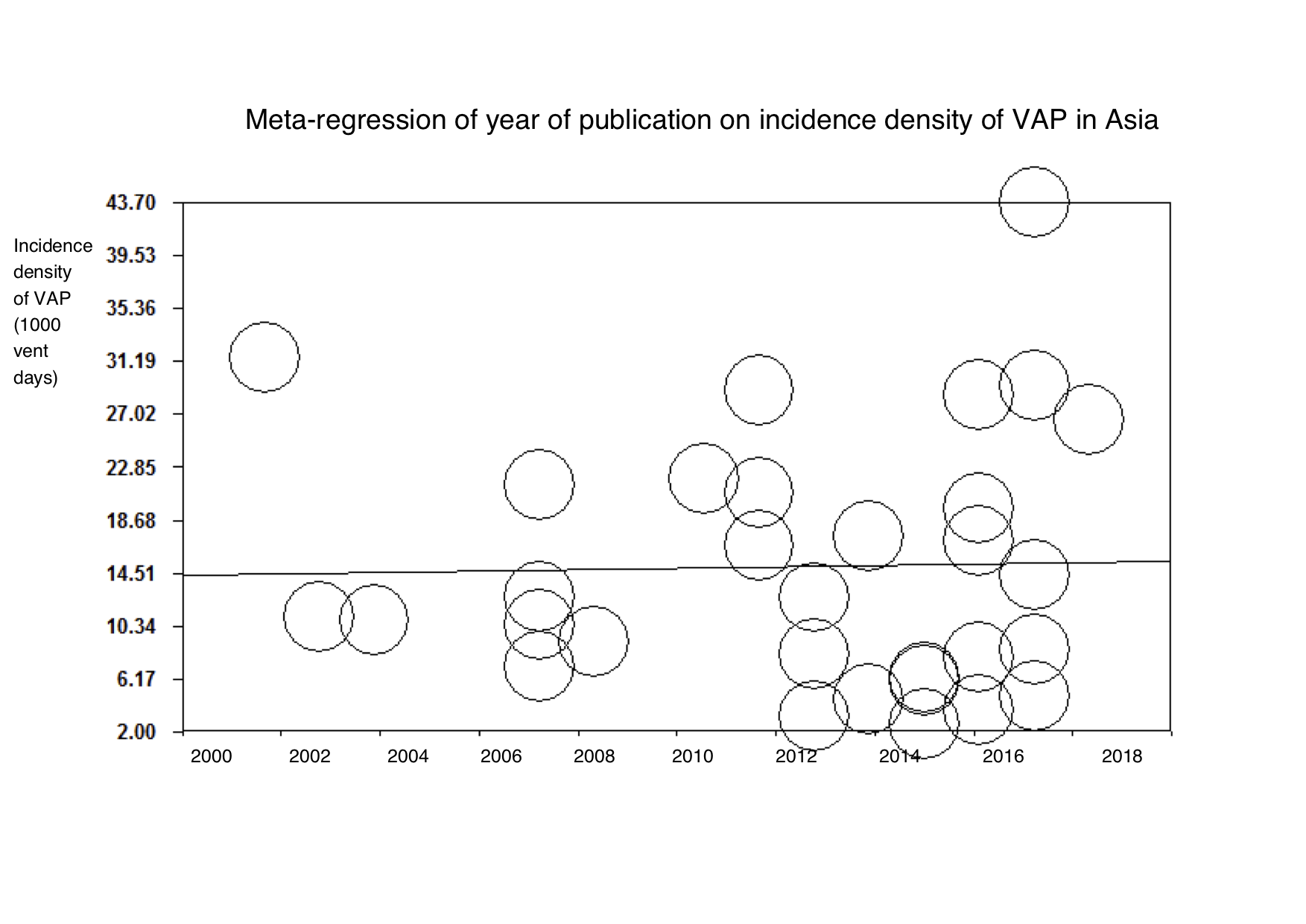

Supplement: Metaregression Year on Incidence Density [file ciy543_suppl_metaregression_year_on_incidence_density.png]

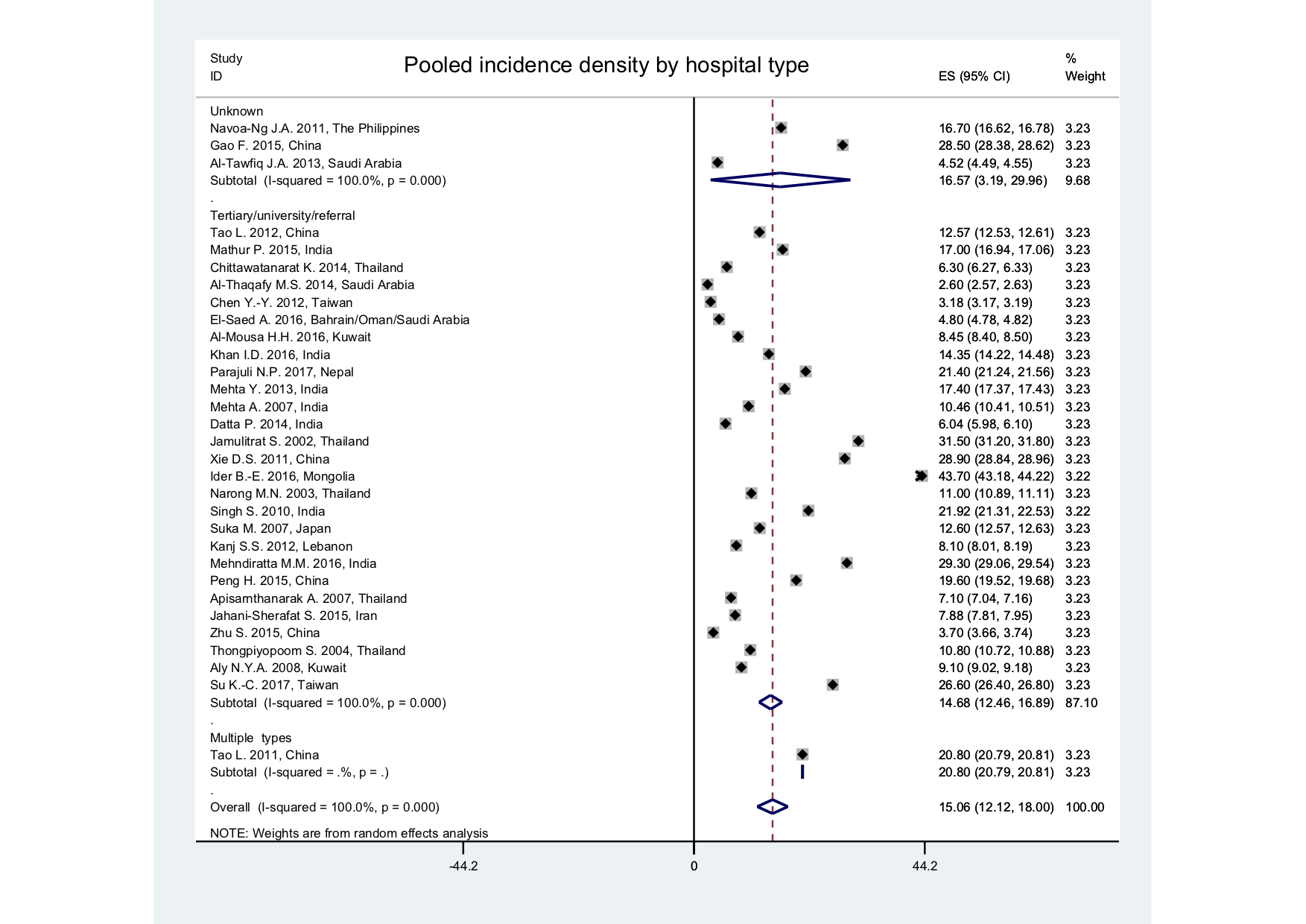

Supplement: Additional Forest Plots of Incidence Density [file ciy543_suppl_additional_forest_plots_of_incidence_density.png]

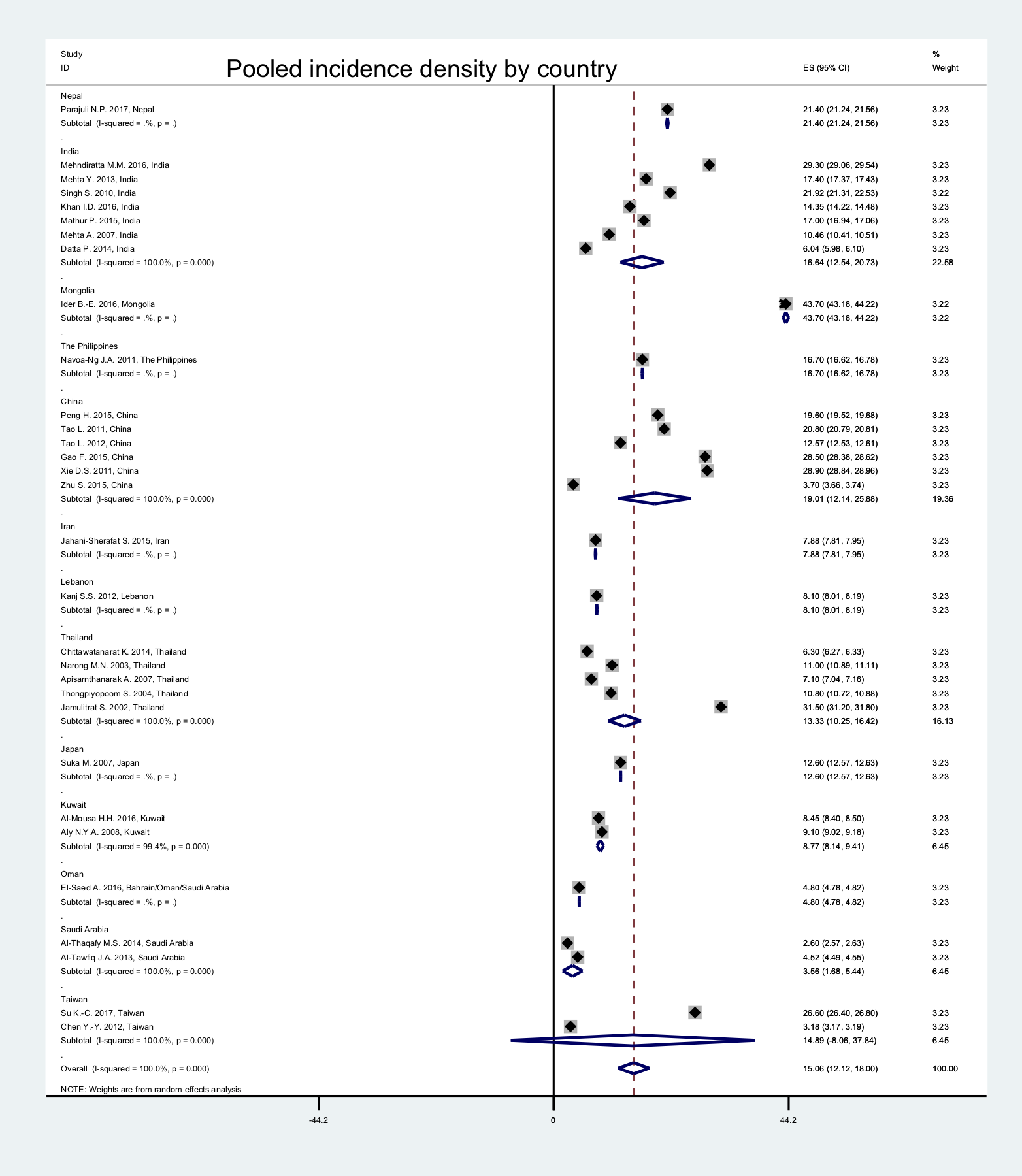

Supplement: Incidence Density Random Forest Plot Country [file ciy543_suppl_incidence_density_random_forest_plot_country.png]

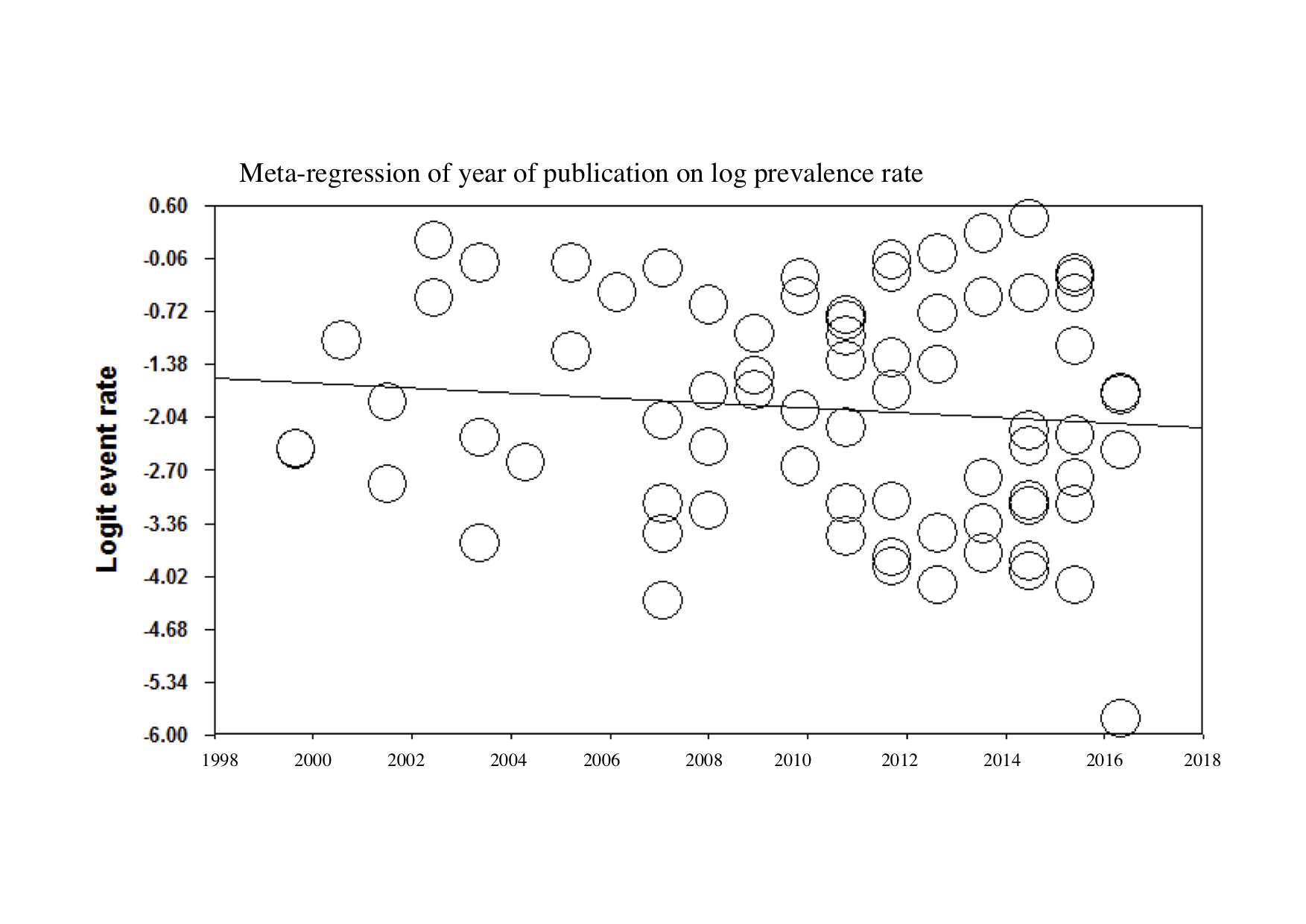

Supplement: Metaregression of Year Published on Prevalence [file ciy543_suppl_metaregression_of_year_published_on_prevalence.png]

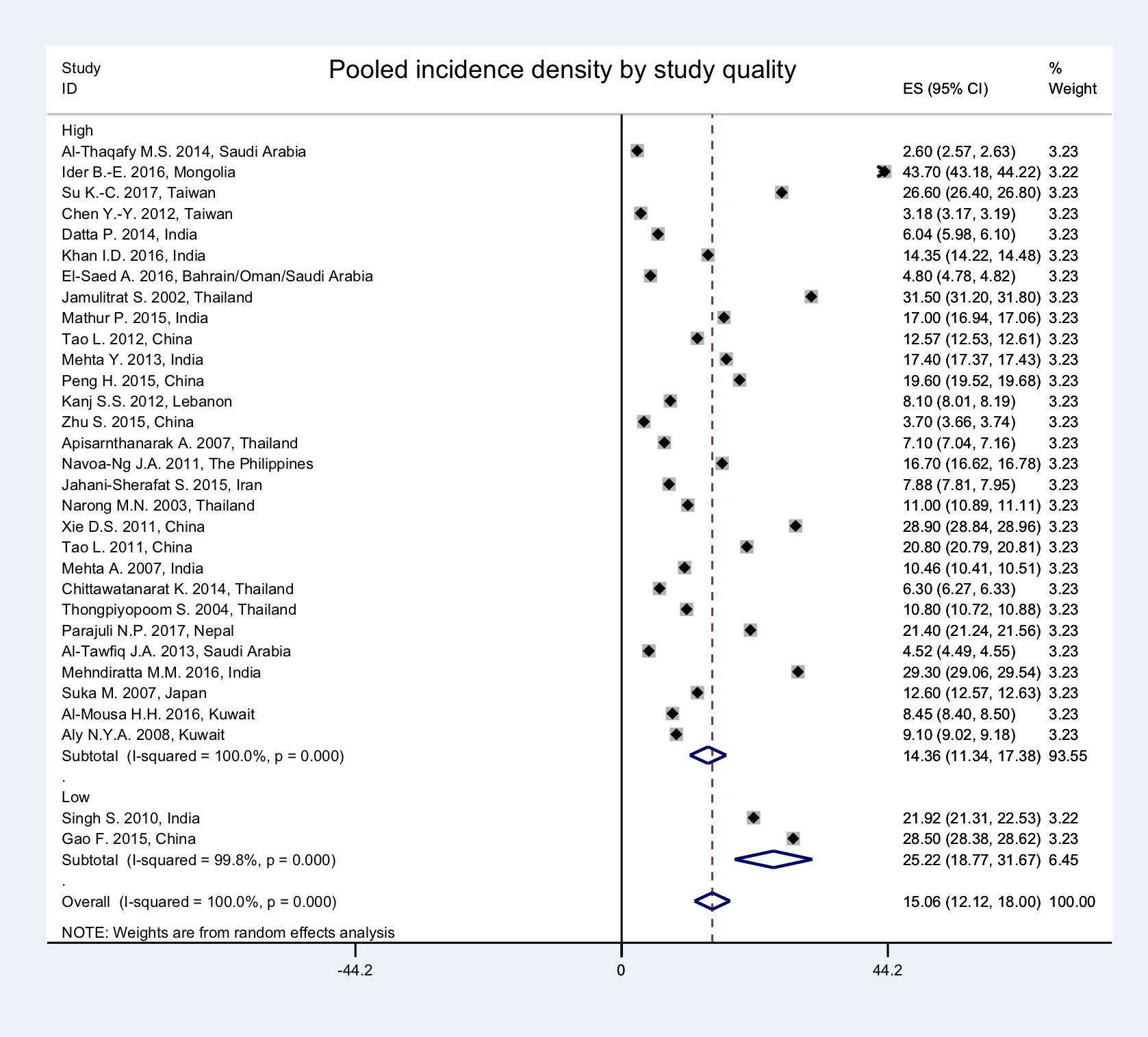

Supplement: Incidence Density Random Forest Plot Qualitygrade [file ciy543_suppl_incidence_density_random_forest_plot_qualitygrade.png]

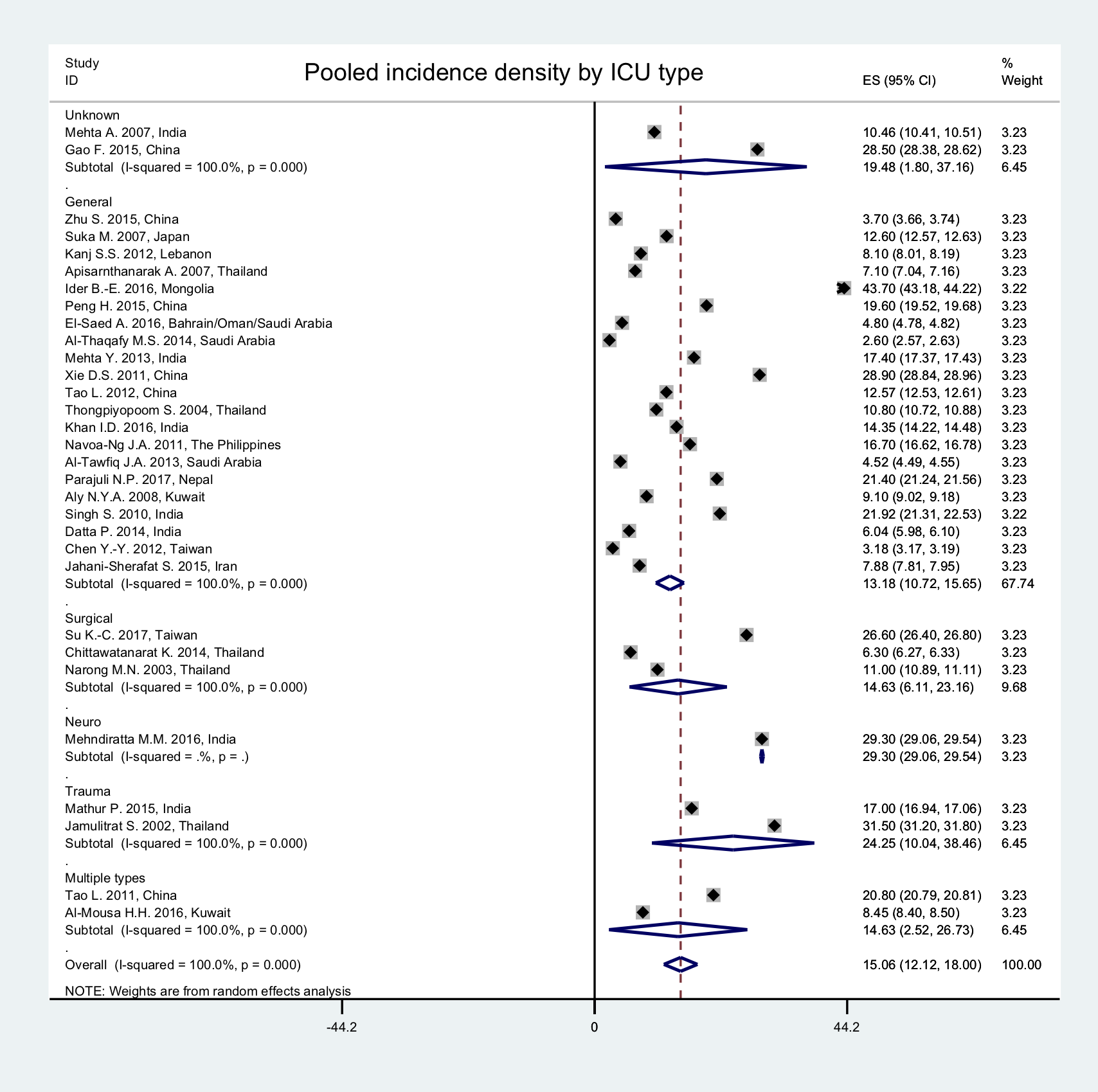

Supplement: Incidence Density Random Forest Plot Icutype [file ciy543_suppl_incidence_density_random_forest_plot_icutype.png]
