# Supplementary material for: A Systematic Review and Meta-analysis of Ventilator-associated Pneumonia in Adults in Asia: An Analysis of National Income Level on Incidence and Etiology
Source: Clin Infect Dis. 2018 Jul 5;68(3):511–8. doi: 10.1093/cid/ciy543 (PMC6336913; doi:10.1093/cid/ciy543)
Supplement: All studies included in SR review [file ciy543_suppl_all_studies_included_in_sr_review.docx]

| **Paper ID** | **Paper Title** | **First Author** | **Year of Publication** | **Publication** |
| --- | --- | --- | --- | --- |
| 2014595982 | Ventilator-associated pneumonia in a teaching hospital in Tehran and use of the Iranian Nosocomial Infections Surveillance software. | Afhami S. | 2012 | Eastern Mediterranean Health Journal. 19 (10) (pp 883-887), 2013. Date of Publication: 2013. |
| 16343637 | Epidemiology, risk factors and outcome of nosocomial infections in a Respiratory Intensive Care Unit in North India. | Agarwal R | 2005 | J Infect. 2006 Aug;53(2):98-105. Epub 2005 Dec 15. |
| 617118996 | Active surveillance of health care associated infections in neurosurgical patients. | Agarwal R | 2017 | Journal of Clinical and Diagnostic Research. 11 (7) (pp DC01-DC04), 2017. Date of Publication: 01 Jul 2017. |
| 97252293 | Treatment of ventilator-associated pneumonia with piperacillin-tazobactum and amikacin vs cefepime and levoß oxacin: A randomized prospective study | Ahmed SM | 2007 | Indian Journal of Critical Care Medicine |
| 2012640931 | The results of a 6-year epidemiologic surveillance for ventilator- associated pneumonia at a tertiary care intensive care unit in Saudi Arabia. | Al-Dorzi H.M. | 2012 | American Journal of Infection Control. 40 (9) (pp 794-799), 2012. Date of Publication: November 2012. |
| 607554501 | Device-associated infection rates, bacterial resistance, length of stay, and mortality in Kuwait: International Nosocomial Infection Consortium findings. | Al-Mousa H.H. | 2016 | American Journal of Infection Control. 44 (4) (pp 444-449), 2016. Date of Publication: 01 Apr 2016. |
| 2013754144 | Reduction and surveillance of device-associated infections in adult intensive care units at a Saudi Arabian hospital, 2004-2011. | Al-Tawfiq J.A. | 2013 | International Journal of Infectious Diseases. 17 (12) (pp e1207-e1211), 2013. Date of Publication: December 2013. |
| 2010474498 | Decreasing ventilator-associated pneumonia in adult intensive care units using the Institute for Healthcare Improvement bundle. | Al-Tawfiq J.A. | 2010 | American Journal of Infection Control. 38 (7) (pp 552-556), 2010. Date of Publication: September 2010. |
| 2014796068 | Association of compliance of ventilator bundle with incidence of ventilator-associated pneumonia and ventilator utilization among critical patients over 4 years. | Al-Thaqafy M.S. | 2014 | Annals of Thoracic Medicine. 9 (4) (pp 221-226), 2014. Date of Publication: 01 Oct 2014. |
| 605808201 | Microbiological pattern of ventilator associated pneumonia | Ali S. | 2015 | Journal of Ayub Medical College, Abbottabad : JAMC. 27 (1) (pp 117-119), 2015. Date of Publication: 01 Jan 2015. |
| 2014269168 | Microbiological and minimum inhibitory concentration study of ventilator-associated pneumonia agents in two university-associated hospital intensive care units in Mazandaran. | Alikhami A. | 2013 | Archives of Clinical Infectious Diseases. 8 (1) (pp 8-13), 2013. Date of Publication: January 2013. |
| 2008387422 | Nosocomial infections in a medical-surgical intensive care unit. | Aly N.Y.A. | 2008 | Medical Principles and Practice. 17 (5) (pp 373-377), 2008. Date of Publication: August 2008. |
| 2007442460 | Effectiveness of an educational program to reduce ventilator-associated pneumonia in a tertiary care center in Thailand: A 4-year study. | Apisarnthanarak A. | 2007 | Clinical Infectious Diseases. 45 (6) (pp 704-711), 2007. Date of Publication: 15 Sep 2007. |
| 2013542387 | Effects of ranitidine and pantoprazole on Ventilator- associated Pneumonia: A randomized double-blind clinical trial. | Bashar F.R. | 2013 | Tanaffos. 12 (2) (pp 16-21), 2013. Date of Publication: 2013. |
| 2012179057 | Application of ventilator care bundle and its impact on ventilator associated pneumonia incidence rate in the adult intensive care unit. | Bukhari S.Z. | 2012 | Saudi Medical Journal. 33 (3) (pp 278-283), 2012. Date of Publication: 2012. |
| 19120729 | Removal of oral secretion prior to position change can reduce the incidence of ventilator-associated pneumonia for adult ICU patients: a clinical controlled trial study | Chao Y-F C | 2007 | journal of clinical nursing |
| 613970263 | A point-prevalence survey of healthcare-associated infection in fifty-two Chinese hospitals. | Chen Y. | 2017 | Journal of Hospital Infection. 95 (1) (pp 105-111), 2017. Date of Publication: 01 Jan 2017. |
| 2012573399 | Surveillance on secular trends of incidence and mortality for device-associated infection in the intensive care unit setting at a tertiary medical center in Taiwan, 2000-2008: A retrospective observational study. | Chen Y.-Y. | 2012 | BMC Infectious Diseases. 12 , 2012. Article Number: 209. Date of Publication: 10 Sep 2012. |
| 2014554510 | Microbiology, resistance patterns, and risk factors of mortality in ventilator-associated bacterial pneumonia in a Northern Thai tertiary-care university based general surgical intensive care unit. | Chittawatanarat K. | 2014 | Infection and Drug Resistance. 7 (pp 203-210), 2014. Date of Publication: 16 Aug 2014. |
| 22749332 | Effect of continuous oral suctioning on the development of ventilator-associated pneumonia: a pilot randomized controlled trial. | Chow MC | 2012 | Int J Nurs Stud. 2012 Nov;49(11):1333-41. doi: 10.1016/j.ijnurstu.2012.06.003. Epub 2012 Jun 28. |
| 2011709308 | High prevalence of multidrug-resistant nonfermenters in hospital-acquired pneumonia in Asia. | Chung D.R. | 2011 | American Journal of Respiratory and Critical Care Medicine. 184 (12) (pp 1409-1417), 2011. Date of Publication: 15 Dec 2011. |
| 2015867228 | Nosocomial infections in the intensive care unit: Incidence, risk factors, outcome and associated pathogens in a public tertiary teaching hospital of Eastern India. | Dasgupta S. | 2015 | Indian Journal of Critical Care Medicine. 19 (1) (pp 14-20), 2015. Date of Publication: 01 Jan 2015. |
| 2014133801 | Health-care-associated infections: Risk factors and epidemiology from an intensive care unit in Northern India. | Datta P. | 2014 | Indian Journal of Anaesthesia. 58 (1) (pp 30-35), 2014. Date of Publication: January -February 2014. |
| 19727346 | Incidence of multidrugresistant organisms causing ventilatorassociated pneumonia in a tertiary care hospital: A nine months' prospective study | Dey A | 2007 | Ann Thorac Med. 2007 Apr;2(2):52-7. doi: 10.4103/1817-1737.32230. |
| 2013511830 | Acinetobacter is the most common pathogen associated with late-onset and recurrent ventilator-associated pneumonia in an adult intensive care unit in Saudi Arabia. | El-Saed A. | 2013 | International Journal of Infectious Diseases. 17 (9) (pp e696-e701), 2013. Date of Publication: September 2013. |
| 609468728 | Ventilator-associated pneumonia rates in critical care units in 3 Arabian Gulf countries: A 6-year surveillance study. | El-Saed A. | 2016 | American Journal of Infection Control. 44 (7) (pp 794-798), 2016. Date of Publication: 01 Jul 2016. |
| 21224971 | A study of ventilator-associated pneumonia: Incidence, outcome, risk factors and measures to be taken for prevention. | Gadani H | 2010 | Indian J Anaesth. 2010 Nov;54(6):535-40. doi: 10.4103/0019-5049.72643. |
| 605525313 | Impact of a bundle on prevention and control of healthcare associated infections in intensive care unit. | Gao F | 2015 | Journal of Huazhong University of Science and Technology. Medical sciences = Hua zhong ke ji da xue xue bao. Yi xue Ying De wen ban = Huazhong keji daxue xuebao. Yixue Yingdewen ban. 35 (2) (pp 283-290), 2015. Date of Publication: 01 Apr 2015. |
| 2011485537 | An audit on ventilator associated pneumonia in the Intensive Care Unit at Teaching Hospital Karapitiya, Galle, Sri Lanka. | Gunaratne A. | 2011 | Anaesthesia, Pain and Intensive Care. 15 (1) (pp 38-41), 2011. Date of Publication: June 2011. |
| 18820195 | Epidemiology of nosocomial infections in medicine intensive care unit at a tertiary care hospital in northern India. | Habibi S | 2008 | Trop Doct. 2008 Oct;38(4):233-5. doi: 10.1258/td.2008.070395. |
| 2013201693 | Device-associated infection rates, device use, length of stay, and mortality in intensive care units of 4 Chinese hospitals: International Nosocomial Control Consortium findings. | Hu B. | 2013 | American Journal of Infection Control. 41 (4) (pp 301-306), 2013. Date of Publication: April 2013. |
| 608756070 | Multicenter study of device-associated infection rates in hospitals of Mongolia: Findings of the International Nosocomial Infection Control Consortium (INICC). | Ider B.-E. | 2016 | American Journal of Infection Control. 44 (3) (pp 327-331), 2016. Date of Publication: 01 Mar 2016. |
| 604584678 | Device-associated infection rates and bacterial resistance in six academic teaching hospitals of Iran: Findings from the International Nocosomial Infection Control Consortium (INICC). | Jahani-Sherafat S | 2015 | Journal of Infection and Public Health. 8 (6) (pp 553-561), 2015. Date of Publication: November 2015. |
| 22754435 | Characterisation of aerobic bacteria isolated from endotracheal aspirate in adult patients suspected ventilator associated pneumonia in a tertiary care center in Mangalore. | Jakribetth RP | 2012 | Saudi J Anaesth. 2012 Apr;6(2):115-9. doi: 10.4103/1658-354X.97022. |
| 23477492 | Trauma severity scoring systems as predictors of nosocomial infection. | Jamulitrat S | 2002 | Infection Control and Hospital Epidemiology. 23 (5) (pp 268-273), 2002. Date of Publication: 2002. |
| 2011261348 | Ventilator-associated pneumonia in Iranian intensive care units. | Japoni A. | 2011 | Journal of Infection in Developing Countries. 5 (4) (pp 286-293), 2011. Date of Publication: April 2011. |
| 2010028694 | Ventilator-associated pneumonia in a tertiary care hospital in india: Incidence and risk factors. | Joseph N.M. | 2009 | Journal of Infection in Developing Countries. 3 (10) (pp 771-777), 2009. Date of Publication: 2009. |
| 2003505500 | Ventilator-Associated Pneumonia at a Tertiary-Care Center in a Developing Country: Incidence, Microbiology, and Susceptibility Patterns of Isolated Microorganisms. | Kanafani Z.A. | 2003 | Infection Control and Hospital Epidemiology. 24 (11) (pp 864-869), 2003. Date of Publication: November 2003. |
| 2012174771 | International nosocomial infection control consortium findings of device-associated infections rate in an intensive care unit of a Lebanese university hospital. | Kanj S.S. | 2012 | Journal of Global Infectious Diseases. 4 (1) (pp 15-21), 2012. Date of Publication: January-March 2012. |
| 610781046 | Annual antibiotic related economic burden of healthcare associated infections; A cross-sectional population based study. | Karkhane M | 2016 | Iranian Journal of Pharmaceutical Research. 15 (2) (pp 605-610), 2016. Date of Publication: Spring 2016. |
| 606140894 | Case-mix, care processes, and outcomes in medically-Ill patients receiving mechanical ventilation in a low-resource setting from Southern India: A prospective clinical case series. | Karthikeyan B. | 2015 | PLoS ONE. 10 (8) (no pagination), 2015. Article Number: e0135336. Date of Publication: 11 Aug 2015. |
| 2010048551 | Ventilator-associated nosocomial pneumonia in intensive care units in Malaysia. | Katherason S.G. | 2009 | Journal of Infection in Developing Countries. 3 (9) (pp 704-710), 2009. Date of Publication: 2009. |
| 23833615 | The effects of an oral care practice on incidence of pneumonia among ventilator patients in ICUs of selected hospitals in Isfahan, 2010. | Khalifehzadeh A | 2010 | Iran J Nurs Midwifery Res. 2012 Mar;17(3):216-9. |
| 617548839 | Device-Associated Healthcare-Associated Infections (DA-HAI) and the caveat of multiresistance in a multidisciplinary intensive care unit. | Khan I.D. | 2016 | Medical Journal Armed Forces India. 73 (3) (pp 222-231), 2017. Date of Publication: July 2017. |
| 1999435227 | Nosocomial infections in the intensive care units at a University Hospital in a developing Country: Comparison with national nosocomial infections surveillance intensive care unit rates. | Khuri-Bulos N.A. | 1999 | American Journal of Infection Control. 27 (6) (pp 547-552), 1999. Date of Publication: 1999. |
| 2013563351 | Risk factors for acquisition of ventilator-associated pneumonia in adult intensive care units. | Lahoorpour F. | 2013 | Pakistan Journal of Medical Sciences. 29 (5) (pp 1105-1107), 2013. Date of Publication: 2013. |
| 2014487023 | Comparison of the efficacy of esomeprazole and famotidine against stress ulcers in a neurosurgical intensive care unit. | Lee T.H. | 2014 | Advances in Digestive Medicine. 1 (2) (pp 50-53), 2014. Date of Publication: June 2014. |
| 2011150562 | Application of a Nano-antimicrobial film to prevent ventilator-associated pneumonia: A pilot study. | Li W. | 2011 | African Journal of Biotechnology. 10 (10) (pp 1926-1931), 2011. Date of Publication: March 2011. |
| 2014340991 | Microscopic examination of intracellular organisms in bronchoalveolar lavage fluid for the diagnosis of ventilator-associated pneumonia: A prospective multi-center study. | Lui C. | 2014 | Chinese Medical Journal. 127 (10) (pp 1808-1813), 2014. Date of Publication: 2014. |
| 612002254 | Ventilator-associated pneumonia: A persistent healthcare problem in Indian Intensive Care Units!. | Mathai A | 2016 | Lung India. 33 (5) (pp 512-516), 2016. Date of Publication: September-October 2016. |
| 25444392 | Incidence and attributable costs of ventilator-associated pneumonia (VAP) in a tertiary-level intensive care unit (ICU) in northern India. | Mathai AS. | 2015 | Journal of Infection and Public Health. 8(2):127-35, 2015 Mar-Apr. |
| 2015667984 | Device-associated infections at a level-1 trauma centre of a developing Nation: Impact of automated surveillance, training and feedbacks. | Mathur P. | 2015 | Indian Journal of Medical Microbiology. 33 (1) (pp 51-62), 2015. Date of Publication: 01 Jan 2015. |
| 608489350 | Ventilators in ICU: A boon or burden. | Mehndiratta M.M | 2016 | Annals of Indian Academy of Neurology. 19 (1) (pp 69-73), 2016. Date of Publication: January 2016. |
| 2007490264 | Device-associated nosocomial infection rates in intensive care units of seven Indian cities. Findings of the International Nosocomial Infection Control Consortium (INICC). | Mehta A. | 2007 | Journal of Hospital Infection. 67 (2) (pp 168-174), 2007. Date of Publication: October 2007. |
| 2014908359 | Effectiveness of a multidimensional approach for prevention of ventilator-associated pneumonia in 21 adult intensive-care units from 10 cities in India: Findings of the International Nosocomial Infection Control Consortium (INICC). | Mehta Y. | 2013 | Epidemiology and Infection. 141 (12) (pp 2483-2491), 2013. Date of Publication: 2013. |
| 11584255 | A randomized clinical trial to compare the effects of a heat and moisture exchanger with a heated humidifying system on the occurrence rate of ventilator-associated pneumonia. | Memish | 2001 | Am J Infect Control. 2001 Oct;29(5):301-5. |
| 10782591 | The incidence and risk factors of ventilator-associated pneumonia in a Riyadh hospital. | Memish | 2000 | Infect Control Hosp Epidemiol. 2000 Apr;21(4):271-3. |
| 617296986 | Economic burden of antibiotic treatment of healthcare-associated infections at a tertiary care hospital ICU in Goa, India. | Misal D.D. | 2017 | Tropical Doctor. 47 (3) (pp 197-201), 2017. Date of Publication: 01 Jul 2017. |
| 2008400988 | Role of quantitative endotracheal aspirate and cultures as a surveillance and diagnostic tool for ventilator associated pneumonia: A pilot study. | Nair S | 2008 | Indian Journal of Medical Sciences. 62 (8) (pp 304-313), 2008. Date of Publication: 01 Aug 2008. |
| 2003323831 | Surgical site infections in patients undergoing major operations in a university hospital: Using standardized infection ratio as a benchmarking tool. | Narong M.N. | 2003 | American Journal of Infection Control. 31 (5) (pp 274-279), 2003. Date of Publication: August 2003. |
| 2011470884 | Device-associated infections rates in adult, pediatric, and neonatal intensive care units of hospitals in the Philippines: International Nosocomial Infection Control Consortium (INICC) findings. | Navoa-Ng J.A. | 2011 | American Journal of Infection Control. 39 (7) (pp 548-554), 2011. Date of Publication: September 2011. |
| 615431797 | Epidemiology of device-associated infections in an intensive care unit of a teaching hospital in Nepal: A prospective surveillance study from a developing country. | Parajuli N.P. | 2017 | American Journal of Infection Control. (no pagination), 2017. Date of Publication: 2017. |
| 2003102443 | Ventilator-associated pneumonia: Incidence, risk factors, outcome, and microbiology. | Pawar M. | 2003 | Journal of Cardiothoracic and Vascular Anesthesia. 17 (1) (pp 22-28), 2003. Date of Publication: February 2003. |
| 611147706 | Health care-associated infections surveillance in an intensive care unit of a university hospital in China, 2010-2014: Findings of International Nosocomial Infection Control Consortium. | Peng H | 2015 | American Journal of Infection Control. 43 (12) (pp e83-e85), 2015. Date of Publication: 2015. |
| 2006426059 | Detection and quantitation of the etiologic agents of ventilator-associated pneumonia in endotracheal tube aspirates from patients in Iran [1]. | Rahbar M. | 2006 | Infection Control and Hospital Epidemiology. 27 (8) (pp 884-885), 2006. Date of Publication: August 2006. |
| 9725229 | Incidence, clinical outcome, and risk stratification of ventilator-associated pneumonia—a prospective cohort study | Rakshit | 2005 | Indian Journal of Critical Care Medicine |
| 2012340371 | Incidence and risk factors for ventilator-associated pneumonia in Kathmandu University Hospital. | Ranjit S. | 2011 | Kathmandu University Medical Journal. 9 (33) (pp 28-31), 2011. Date of Publication: January-March 2011. |
| 24050082 | Causative agents and resistance among hospital-acquired and ventilator-associated pneumonia patients at Srinagarind Hospital, northeastern Thailand. | Reechaipichitkul W. | 2013 | Southeast Asian Journal of Tropical Medicine and Public Health. 44 (3) (pp 490-502), 2013. Date of Publication: May 2013. |
| 11771082 | The Incidence of Nosocomial Infection in the Intensive Care Unit, Hospital Universiti Kebangsaan Malaysia: ICU-acquired Nosocomial Infection Surveillance Program 1998..1999 | Rozaidi SW | 2001 | Med J Malaysia Val 56 No 2 June 200 I |
| 612060470 | Oral health and ventilator-associated pneumonia among critically ill patients: a prospective study. | Saensom D | 2016 | Oral Diseases. 22 (7) (pp 709-714), 2016. Date of Publication: 01 Oct 2016. |
| 25183978 | Effect of intermittent subglottic secretion drainage on ventilator-associated pneumonia: A clinical trial. | Safdari R | 2014 | Iranian Journal of Nursing and Midwifery Research. 19(4):376-80, 2014 Jul. |
| 2014074705 | Determinants of ventilator associated pneumonia and its impact on prognosis: A tertiary care experience. | Saravu K. | 2013 | Indian Journal of Critical Care Medicine. 17 (6) (pp 337-342), 2013. Date of Publication: 2013. |
| 200933080 | Infection control education: Impact on ventilator-associated pneumonia rates in a public sector intensive care unit in Pakistan. | Siddiqui S.Z. | 2009 | Transactions of the Royal Society of Tropical Medicine and Hygiene. 103 (8) (pp 807-811), 2009. Date of Publication: August 2009. |
| 2010618146 | Surveillance of device-associated infections at a teaching hospital in rural Gujarat - India. | Singh S | 2010 | Indian Journal of Medical Microbiology. 28 (4) (pp 342-347), 2010. Date of Publication: October 2010. |
| 1999327560 | Hospital acquired pneumonia in the Medical Intensive Care Unit - A prospective study. | Stebbings AEL | 1999 | Singapore Medical Journal. 40 (8) (pp 508-512), 1999. Date of Publication: 1999. |
| 617606395 | A simplified prevention bundle with dual hand hygiene audit reduces early-onset ventilator-associated pneumonia in cardiovascular surgery units: An interrupted time-series analysis. | Su K.-C. | 2017 | PLoS ONE. 12 (8) (no pagination), 2017. Article Number: e0182252. Date of Publication: August 2017. |
| 2007153777 | Incidence and outcomes of ventilator-associated pneumonia in Japanese intensive care units: The Japanese nosocomial infection surveillance system. | Suka M | 2007 | Infection Control and Hospital Epidemiology. 28 (3) (pp 307-313), 2007. Date of Publication: March 2007. |
| 2012276117 | A survey on microorganisms and their sensitivity by E-test in ventilator-associated pneumonia at toxicological-intensive care unit of Loghman-Hakim hospital. | Talaie H | 2010 | Acta Biomedica. 81 (3) (pp 210-216), 2010. Date of Publication: 2010. |
| 2008089553 | Randomized controlled trial and meta-analysis of oral decontamination with 2% chlorhexidine solution for the prevention of ventilator-associated pneumonia. | Tantipong H. | 2008 | Infection Control and Hospital Epidemiology. 29 (2) (pp 131-136), 2008. Date of Publication: February 2008. |
| 2011592127 | Device-associated infection rates in 398 intensive care units in Shanghai, China: International Nosocomial Infection Control Consortium (INICC) findings. | Tao L | 2011 | International Journal of Infectious Diseases. 15 (11) (pp e774-e780), 2011. Date of Publication: November 2011. |
| 2012597870 | Impact of a multidimensional approach on ventilator-associated pneumonia rates in a hospital of Shanghai: Findings of the International Nosocomial Infection Control | Tao L | 2012 | Journal of Critical Care. 27 (5) (pp 440-446), 2012. Date of Publication: October 2012. |
| 2004376861 | Device-associated infections and patterns of antimicrobial resistance in a medical-surgical intensive care unit in a University Hospital in Thailand. | Thongpiyopoom S. | 2004 | Journal of the Medical Association of Thailand. 87 (7) (pp 819-824), 2004. Date of Publication: July 2004. |
| 17711140 | Using a collaborative to reduce ventilator-associated pneumonia in Thailand. | Unahalekhaka A | 2007 | Jt Comm J Qual Patient Saf. 2007 Jul;33(7):387-94. |
| 12171848 | Quantitative Culture of Endotracheal Aspirates in the Diagnosis of Ventilator- Associated Pneumonia in Patients With Treatment Failure* | Wu CL | 2002 | Chest. 2002 Aug;122(2):662-8. |
| 2011381583 | Ventilator-associated pneumonia in intensive care units in Hubei Province, China: A multicentre prospective cohort survey. | Xie D.S. | 2011 | Journal of Hospital Infection. 78 (4) (pp 284-288), 2011. Date of Publication: August 2011. |
| 609609254 | Effect of probiotics on the incidence of ventilator-associated pneumonia in critically ill patients: a randomized controlled multicenter trial. | Zeng J. | 2016 | Intensive Care Medicine. 42 (6) (pp 1018-1028), 2016. Date of Publication: 01 Jun 2016. |
| 2015030354 | Care bundle for ventilator-associated pneumonia in a medical intensive care unit in Northern Taiwan. | Zeng W-P | 2015 | Journal of Medical Sciences (Taiwan). 35 (2) (pp 68-73), 2015. Date of Publication: 01 Mar 2015. |
| 608515557 | The clinical impact of ventilator-associated events: A prospective multi-center surveillance study. | Zhu S. | 2015 | Infection Control and Hospital Epidemiology. 36 (12) (pp 1388-1395), 2015. Date of Publication: 2015. |
